# Supplementary material for: Drug-Induced Resistance and Phenotypic Switch in Triple-Negative Breast Cancer Can Be Controlled via Resolution and Targeting of Individualized Signaling Signatures
Source: Cancers (Basel). 2021 Oct 6;13(19):5009. doi: 10.3390/cancers13195009 (PMC8507629; doi:10.3390/cancers13195009)
Supplement: Supplementary file 1 [file cancers-13-05009-s001.zip › cancers-1398648-supplementary-final/cancers-1398648-supplementary-final.pdf]

Supplementary Materials

Drug-Induced Resistance and Phenotypic Switch in Triple Negative Breast Cancer Can Be Controlled via Resolution and Targeting of Individualized Signaling Signatures

Swetha Vasudevan, Ibukun A. Adejumobi, Heba Alkhatib, Sangita Roy Chowdhury, Shira Stefansky, Ariel M. Rubinstein and Nataly Kravchenko-Balasha

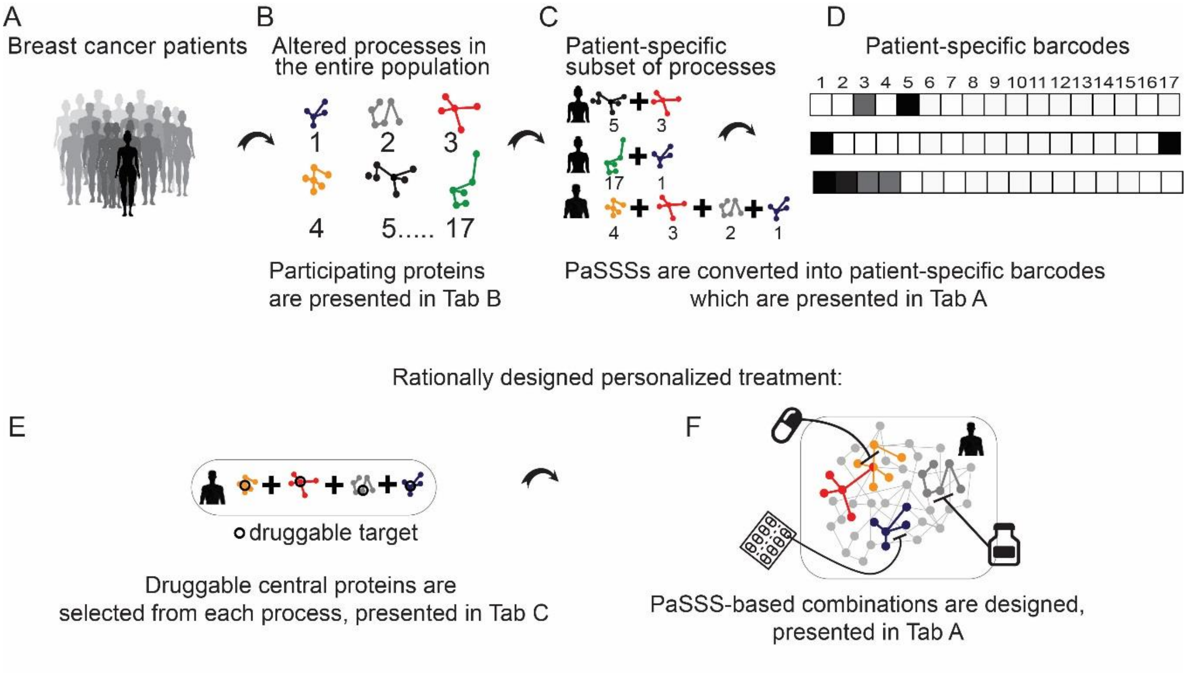

Figure S1. Flowchart for the use of the PaSSS-based data (**A**) Following the acquisition of cancer tissues and proteomics profiling, Surprisal analysis is utilized in order to uncover the altered (unbalanced processes) in which all molecules (e.g., proteins) undergo coordinated deviations from the reference (steady) state (**B**, Tab B in Table S1). For more details see reference 18 in the main text. Next, a patient-specific set of significant unbalanced processes is determined (**C**, Tab A in Table S1) for each patient and converted into an individualized barcode (**D**, Tab A in Table S1). Each tumor-specific unbalanced network is examined, aiming to identify and verify experimentally the major hubs whose blockage is expected to lead to a collapse of the patient-specific unbalanced network (**E**, Tab C in Table S1). Finally, a tumor-specific combination of targeted therapies is tailored to every patient (**F**, Tab C in Table S1). (Abbreviations: PaSSS—Patient-specific signaling signature).

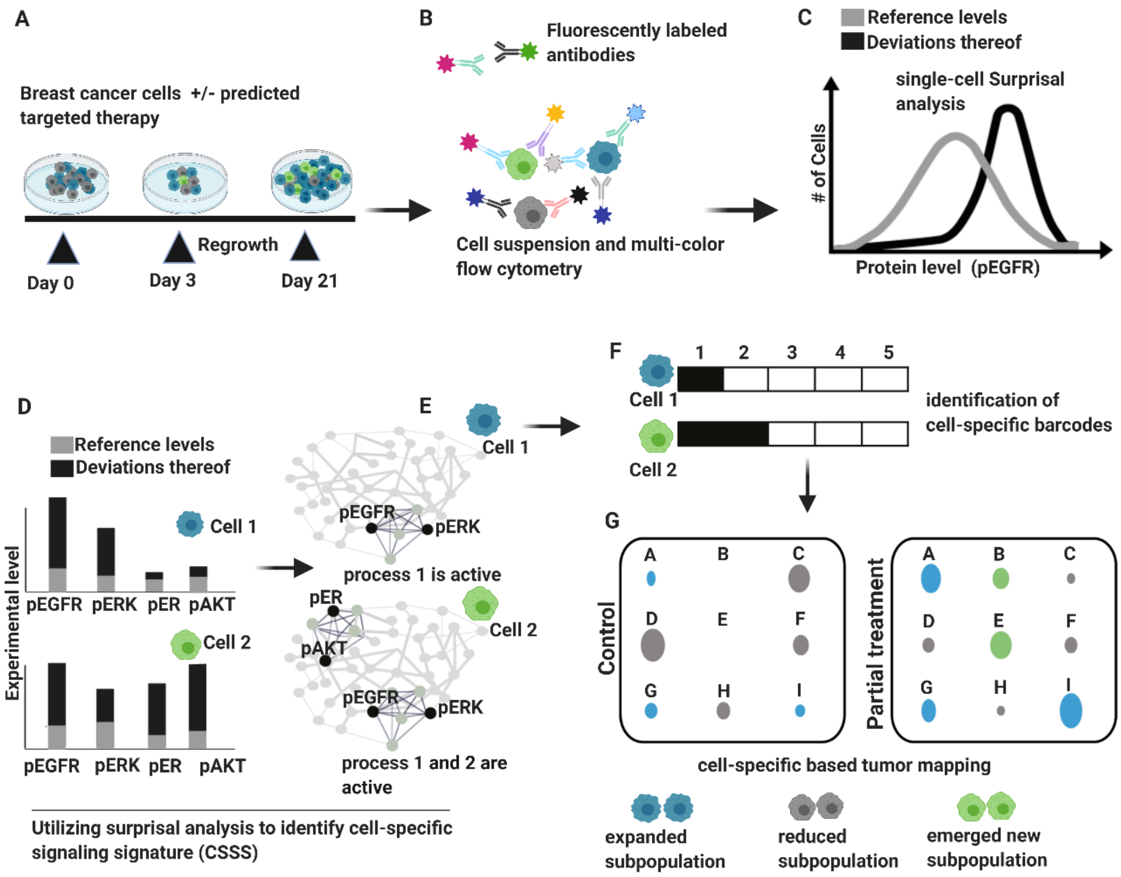

Figure S2. Scheme of the algorithm of the single-cell surprisal analysis. (**A**) TNBC cells were treated with drug combinations as predicted using PaSSS analysis for 72 h and were allowed to regrow at various time points; (**B**) Single-cell suspension was labeled with fluorescently tagged antibodies against central targets as indicated in the main text; (**C,D**) Single-cell surprisal analysis identifies the distribution of protein expression levels at reference state and deviation thereof (Methods); (**E**) Proteins that similarly deviate from the reference state are grouped into altered subnetworks (unbalanced processes); (**F**) Several processes may be active in each cell, thus a cell-specific signaling signature (CSSS) is assigned to each cell which is then transformed into a cell-specific barcode; (**G**) Cells sharing the same barcode are organized into a subpopulation. (Abbreviations: TNBC—Triple Negative Breast Cancer, pEGFR—phospho Epidermal Growth Factor Receptor, pER—phospho Estrogen Receptor, pAKT—phospho Protein kinase B, pERK—phospho Extracellular signal-regulated kinase, CSSS—Cell-specific signaling signature, PaSSS—Patient-specific signaling signature).

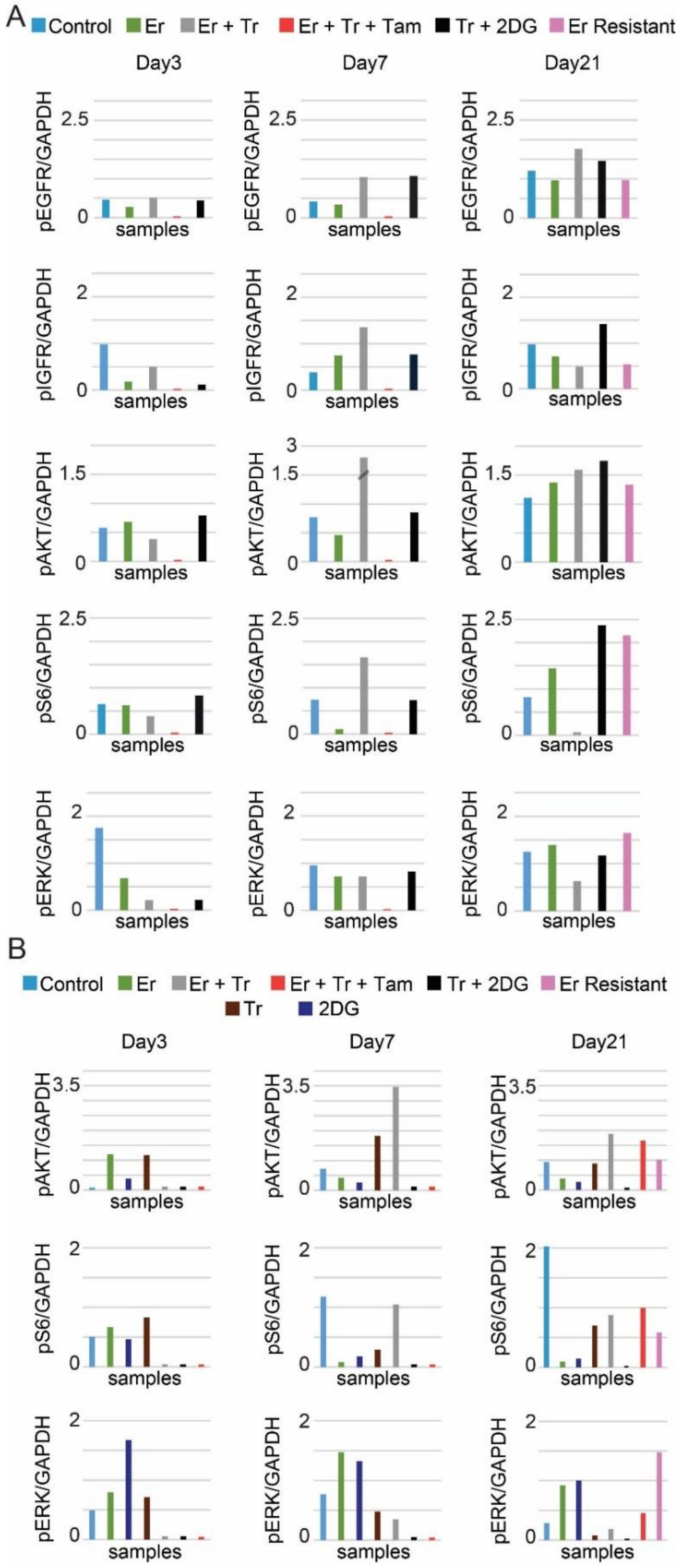

Figure S3. Quantification of the western blot analysis shown in Figure 3 of the main text. (**A**) Column graphs show the normalized protein expression levels of MDA-MB-468 samples treated with different therapies. The intensity of each protein is normalized to the intensity of GAPDH; (**B**) Column graphs show the normalized protein expression levels of MDA-MB-231 samples treated with different therapies. The intensity of each protein is normalized to the total lane intensity obtained by GAPDH. (Abbreviations: Er—Erlotinib, Tr—Trametinib, 2DG—2 deoxyglucose, Tam—Tamoxifen, pEGFR—phospho Epidermal Growth Factor Receptor, pIGFR—phospho Insulin Growth Factor Receptor, pAKT—phospho Protein kinase B, pS6—phospho Ribosomal protein S6, GAPDH—Glyceraldehyde 3-phosphate dehydrogenase, pERK—phospho Extracellular signal-regulated kinase).

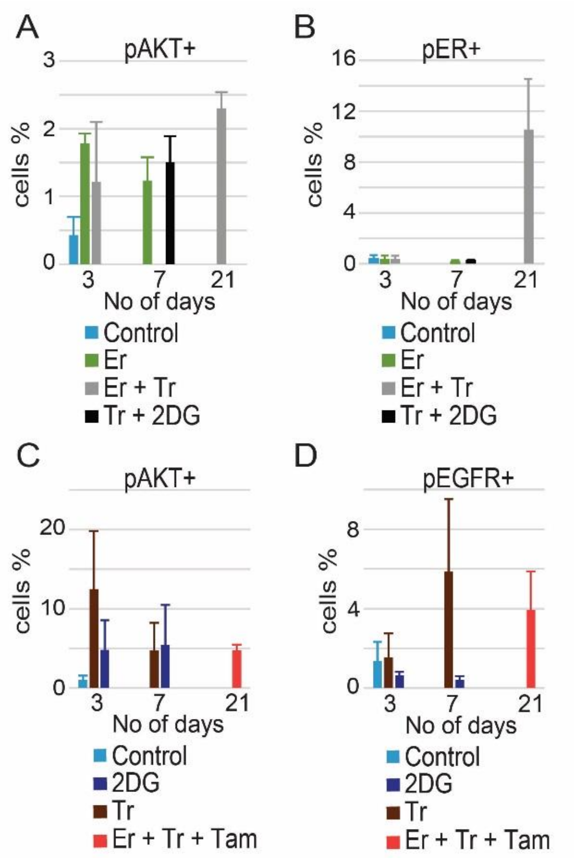

Figure S4. Percentage of the cells harboring pAKT+ and pER+ processes in MDA-MB-468 and MDA-MB-231 cells. (**A,B**) Percentage of the cells harboring pAKT+ and pER+ processes was calculated using results from at least two independent experiments for each time point in MDA-MB-468 cells. The results are mean ± S.E.; (**C,D**) Percentage of the cells harboring pAKT+ and pEGFR+ was calculated using results from at least two independent experiments for each time point in MDA-MB-231 cells. The results are mean ± S.E.; Quantification of the processes and subpopulations was performed using at least ~30,000 cells from each condition, which were obtained from at least 3 wells. (Abbreviations: Er—Erlotinib, Tr—Trametinib, 2DG—2 deoxyglucose, Tam—Tamoxifen, pEGFR—phospho Epidermal Growth Factor Receptor, pAKT—phospho Protein kinase B, pER—phospho Estrogen Receptor, S.E.—standard error, + (e.g., pAkt+) indicates the cells which are positive for a particular protein (express significantly) as indicated.

1. Original Western Blots

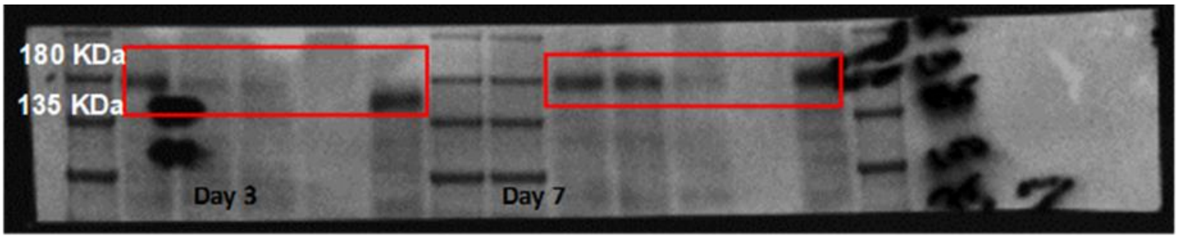

Figure S5. Original western blot for pEGFR for day 3 and 7 in Figure 3E.

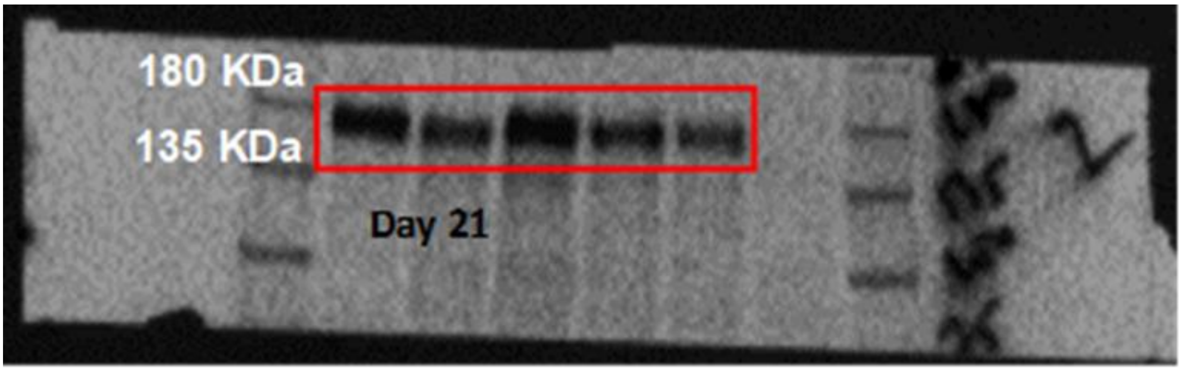

Figure S6. Original western blot for pEGFR for day 21 in Figure 3E.

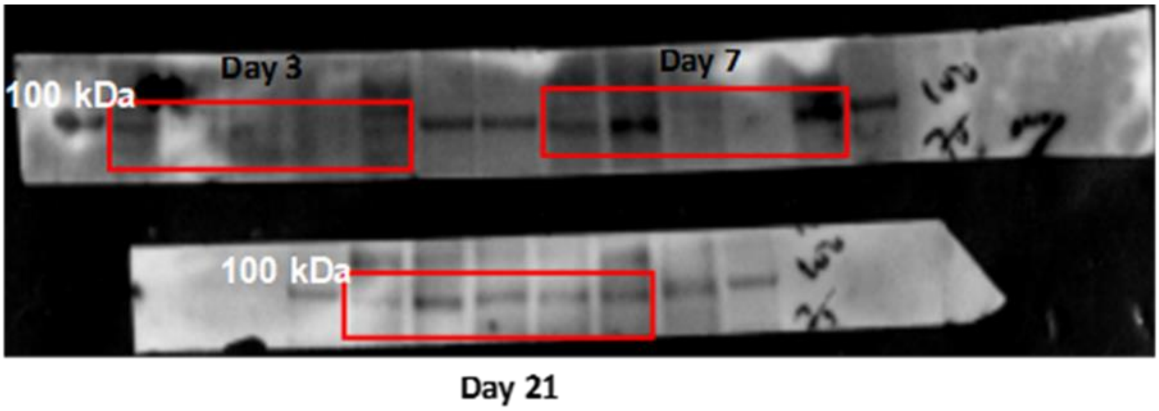

Figure S7. Original western blot for pIGFR in Figure 3E.

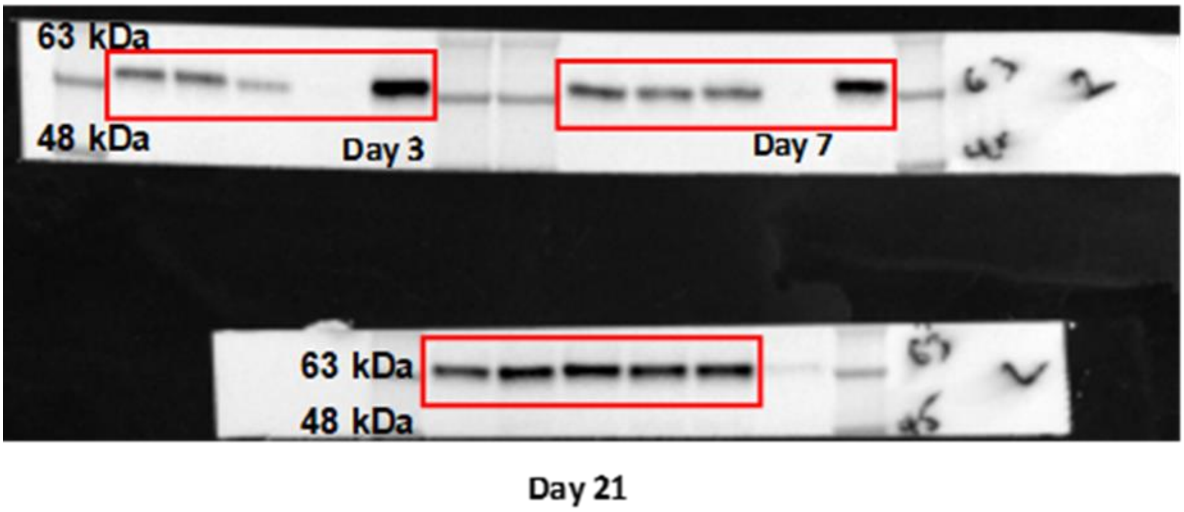

Figure S8. Original western blot for pAKT in Figure 3E.

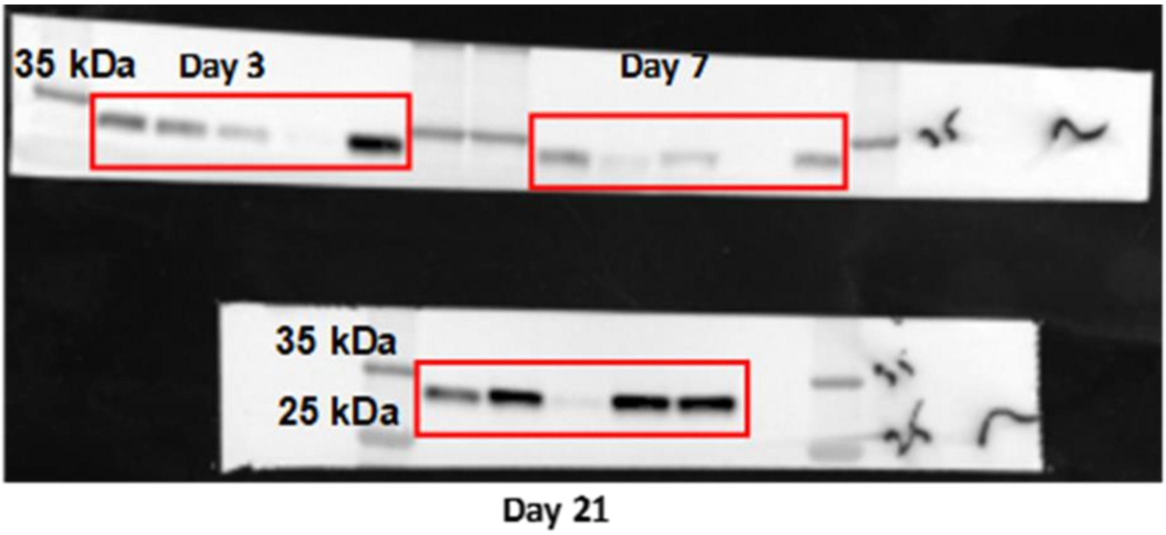

Figure S9. Original western blot for pS6 in Figure 3E.

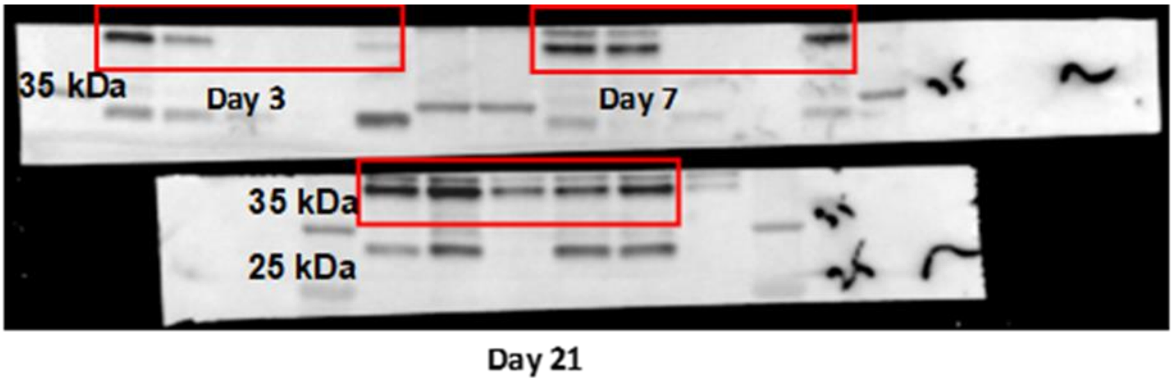

Figure S10. Original western blot for pERK in Figure 3E.

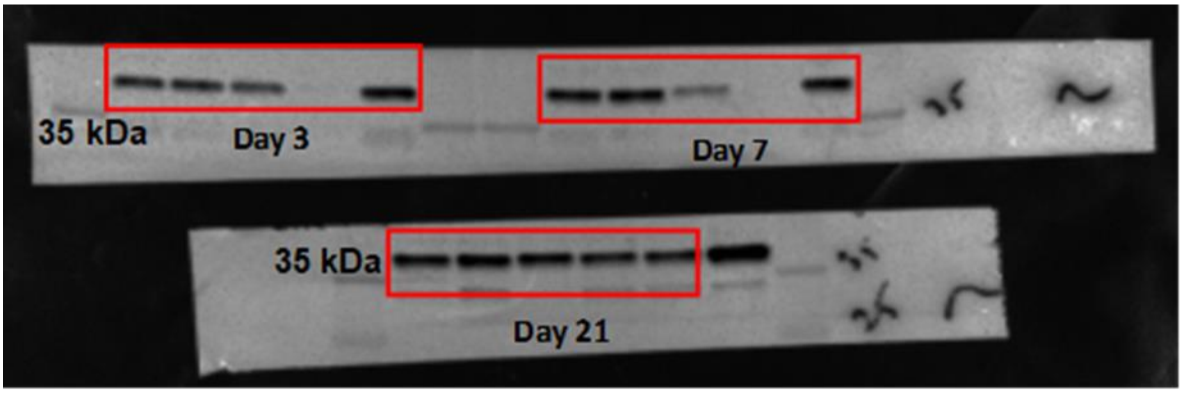

Figure S11. Original western blot for GAPDH in Figure 3E.

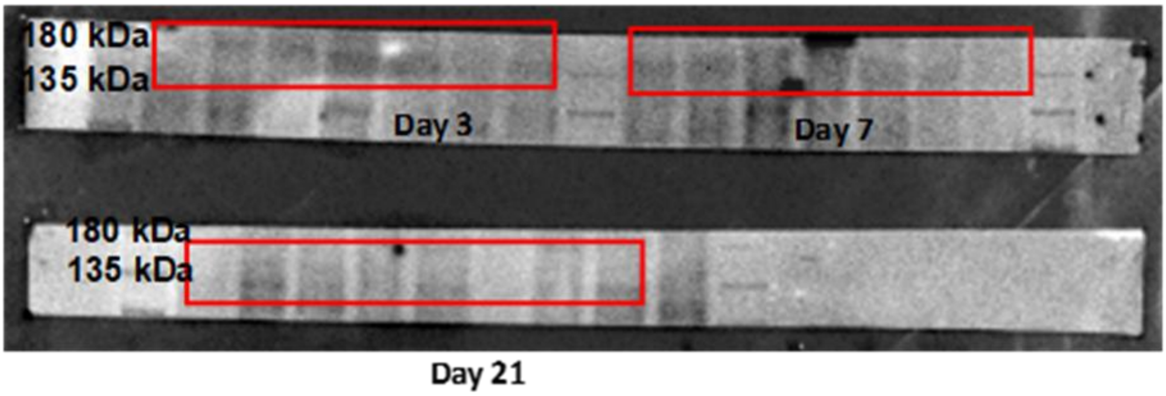

Figure S12. Original western blot for pEGFR in Figure 3F.

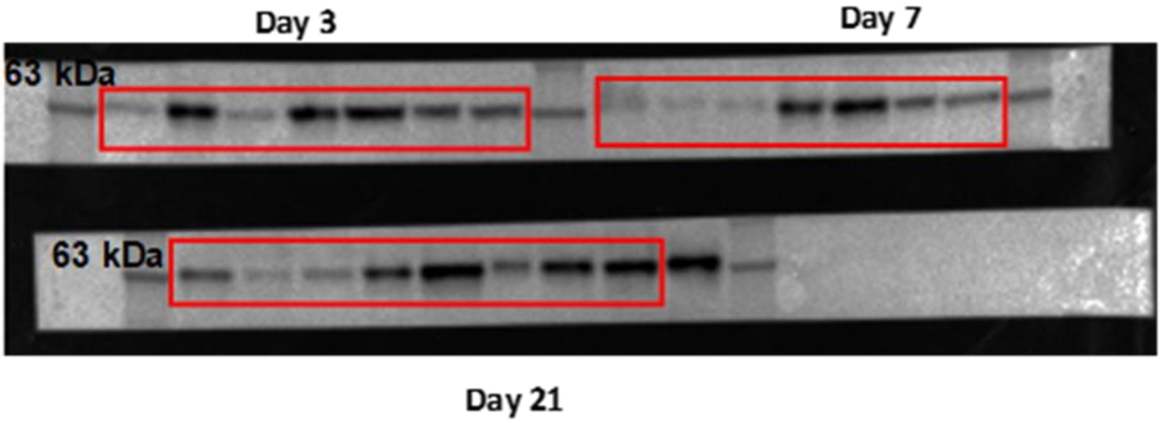

Figure S13. Original western blot for pAKT in Figure 3F.

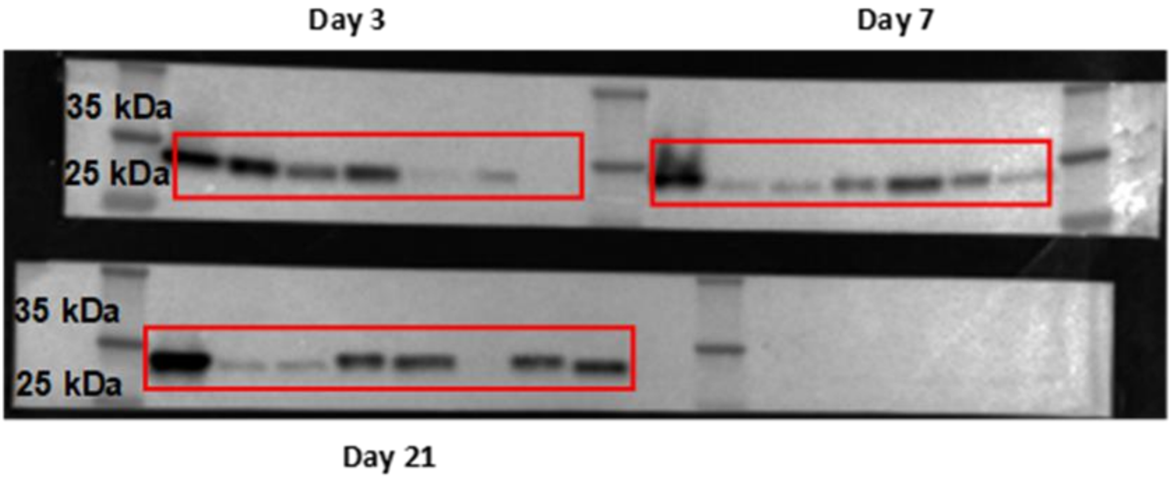

Figure S14. Original western blot for pS6 in Figure 3F.

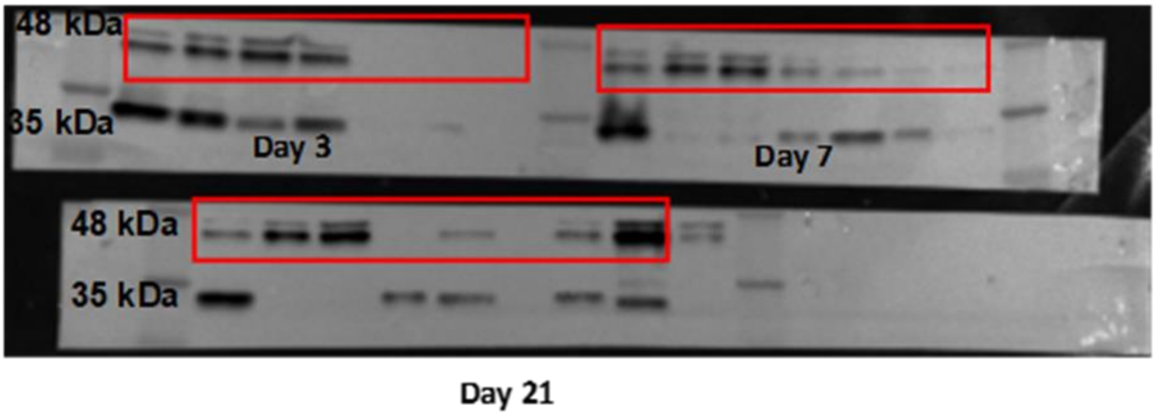

Figure S15. Original western blot for pERK in Figure 3F.

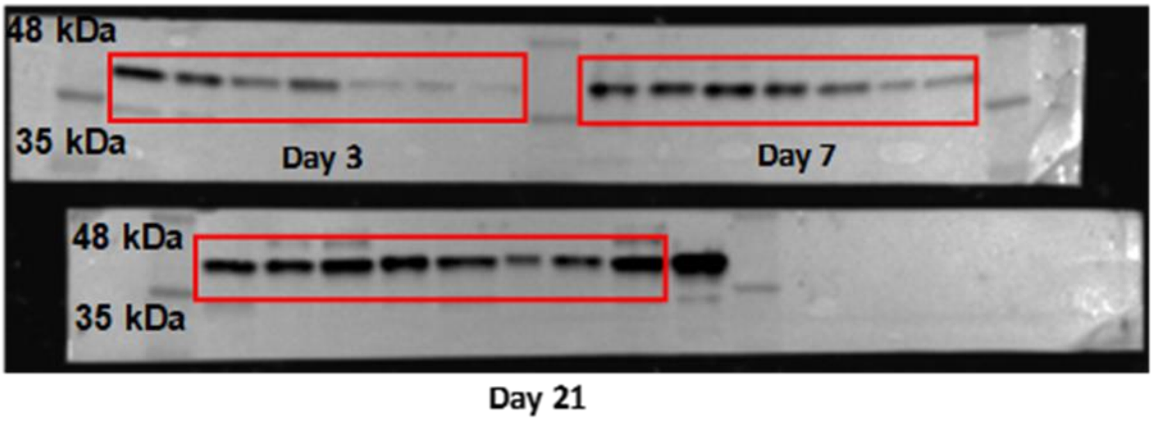

Figure S16. Original western blot for GAPDH in Figure 3F.

© 2021 by the authors. Licensee MDPI, Basel, Switzerland. This article is an open access article distributed under the terms and conditions of the Creative Commons Attribution (CC BY) license (http://creativecommons.org/licenses/by/4.0/).
